# Physically stressed bees expect less reward in an active choice judgement bias test

**DOI:** 10.1098/rspb.2024.0512

**Published:** 2024-10-09

**Authors:** Olga Procenko, Jenny C. A. Read, Vivek Nityananda

**Affiliations:** ^1^ Biosciences Institute, Newcastle University, Henry Wellcome Building, Framlington Place, Newcastle upon Tyne NE2 4HH, UK

**Keywords:** bumblebee, judgment bias, emotion, signal detection theory, drift diffusion, pessimism

## Abstract

Emotion-like states in animals are commonly assessed using judgment bias tests that measure judgements of ambiguous cues. Some studies have used these tests to argue for emotion-like states in insects. However, most of these results could have other explanations, including changes in motivation and attention. To control for these explanations, we developed a novel judgment bias test, requiring bumblebees to make an active choice indicating their interpretation of ambiguous stimuli. Bumblebees were trained to associate high or low rewards, in two different reward chambers, with distinct colours. We subsequently presented bees with ambiguous colours between the two learnt colours. In response, physically stressed bees were less likely than control bees to enter the reward chamber associated with high reward. Signal detection and drift diffusion models showed that stressed bees were more likely to choose low reward locations in response to ambiguous cues. The signal detection model further showed that the behaviour of stressed bees was explained by a reduction in the estimated probability of high rewards. We thus provide strong evidence for judgement biases in bees and suggest that their stress-induced behaviour is explained by reduced expectation of higher rewards, as expected for a pessimistic judgement bias.

## 1. Introduction

The presence of emotions in non-human animals is much debated and can have important societal implications for how we treat animals and assess their welfare. Most research on animal emotions has focused on vertebrates [1,2]. However, some research has investigated emotion-like states in invertebrates [3]. In insects, fruit flies have been used as model systems to investigate neuropsychiatric disorders [4] or states resembling anxiety [5] and fear [6]. More recently, studies have investigated insect emotion-like states based on their effects on cognition [7–11]. These experiments use judgement bias tests, which were developed in animal welfare research to infer animal emotional states [7–11]. The tests are based on the idea that emotions can bias information processing [12]. For example, people experiencing anxiety or depression are more likely to make pessimistic judgments and interpret ambiguous information negatively [13]. To assess their relative reactions to ambiguous stimuli, individual animals are first trained to associate one stimulus with a ‘good’ outcome like a reward, and another with a ‘bad’ or less-positive outcome, like lower or no rewards, or a punishment. Some animals are then subjected to an intervention—either stressful (e.g. poor housing) or positive (e.g. unexpected reward)—while others are unmanipulated and serve as controls. The animals are then tested with ambiguous stimuli designed to be midway between the stimuli indicating good and bad outcomes. If animals experiencing the intervention are more likely than controls to respond as if they expect good or bad outcomes, then it is considered to have made them more optimistic or pessimistic, respectively. These results have often been interpreted as evidence for emotion-like states in animals [14].

Judgement bias tests have been used in five insect studies [7–11]. In some of them, insects learned to respond to specific odours and not to others [3–5]. Physical agitation subsequently reduced their response to ambiguous odours compared with control insects. In other studies, bees learned to associate one colour with a reward and another colour with no reward [7,11]. After encountering an unexpected reward of sucrose solution, the bees were quicker [7] or more likely [11] to fly towards ambiguous colours.

However, four of these five studies [7–9,11] used a go/no-go paradigm, where an animal responds to a positive stimulus (‘go’) and suppresses responses to a negative stimuli (‘no-go’). Subsequently, the latency to approach, or proportion of ‘go’ responses to, the test stimuli was used to infer an animal’s state. This paradigm has been used in numerous studies across different taxa [14], but the measures in this paradigm may be influenced by factors other than cognitive biases. For example, changes in latency or the proportion of ‘go’ responses to ambiguous stimuli could reflect changes in motivation, arousal or attention [15,16]. While motivation and arousal do contribute to emotion-like states, they are different from judgement biases and the evidence for the latter could be strengthened in insects.

The likelihood of confounds can be reduced using an active choice judgment bias test [10,17,18]. This paradigm requires the animal to make an active choice between two alternative responses. Animals might, for example, learn to move to one location in response to one stimulus and another location when they see another. Since the animal must make a choice, this type of test eliminates the possible confounding factors of the go/no-go paradigm, increasing validity and ease of interpretation.

We therefore used an active choice judgment bias test to rigorously assess judgement biases in bumblebees (*Bombus terrestris*). Bees had to choose between two rewarding locations depending on the stimulus displayed, clearly signalling their judgement when faced with ambiguous stimuli by moving to one of the two locations. To induce negative states, we used two types of manipulations simulating predatory attacks—shaking, and trapping by a robotic arm. These manipulations have previously been shown to be associated with cognitive and physiological changes [7,8]. Using two stressors allowed us to ask how generalizable the cognitive impact would be.

Critically, one location in our experiment always contained a high concentration of sucrose (or water), while the other location contained a lower concentration sucrose reward (or water). We hypothesized that stressed bees would be less likely to approach high-reward locations compared with control bees, indicating a judgement bias. If stress instead impaired motivation or attention rather than judgement as has been previously argued [19], we would expect bees to fail to make choices or to respond to the stimuli. Shortened choice latencies have also been previously used as an indicator of optimism in bees [7]. We therefore examined the choice latencies in our experiment. We predicted that conversely, if the stressed bees had pessimistic biases, they would have increased choice latencies. Finally, to further understand the mechanisms underlying our behavioural results, we applied drift diffusion and signal detection modelling frameworks to the data. We used these frameworks to test whether judgement biases in bees could be explained by a change in the estimated probability of a reward.

## 2. Material and methods

### (a) Animals and experimental set-up

All experiments were run on female worker bumblebees (*B. terrestris*) obtained from a commercial supplier (Koppert, UK). We transferred the bumblebees to one chamber of a bipartite plastic nest box (28.0 × 16.0 × 12.0 cm^3^). We covered the other chamber of the nest box with cat litter to allow bees to discard refuse. The nest box was connected via a transparent acrylic tunnel (56.0 × 5.0 × 5.0 cm^3^) to a flight arena (110.0 × 61.0 × 40.0 cm^3^) with an ultraviolet (UV)-transparent Plexiglas^®^ lid and lit by a lamp (HF-P 114-35 TL5 ballast, Philips, The Netherlands) fitted with daylight fluorescent tubes (Osram, Germany). When not part of an experiment, bees were fed with ~3 g of commercially obtained pollen daily (Koppert BV, The Netherlands) and provided sucrose solution (20% w/w) ad libitum. Although invertebrates do not fall under the Animals (Scientific Procedures) Act, 1986 (ASPA), the experimental design and protocols were developed incorporating the 3Rs principles—replacement, reduction and refinement (http://www.nc3rs.org.uk/). The housing, maintenance and experimental procedures used were non-invasive.

**Figure 1.**
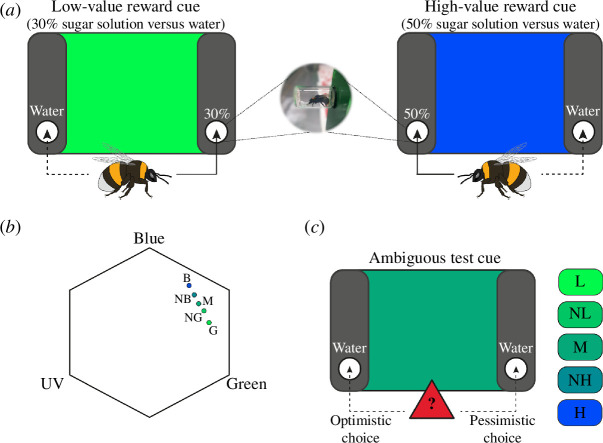
Experimental protocol. (*a*) Bees were trained to associate two colours, green and blue, presented on an LED screen with different sugar rewards at different locations. The figure depicts a training scenario with green indicating a low reward (30% sucrose solution) in the right chamber and blue indicating a high reward (50% sucrose solution) in the left chamber. The association between colour, reward and location was counterbalanced across trials. Further details in the text. (*b*) Cue colours plotted in bee colour space (colour cue: B, blue; NB, near blue; M, medium; NG, near green; G, green). The three vertices correspond to maximum excitation of photoreceptors sensitive to blue, green and UV light. The distance from the centre to any vertex is 1 and the distance between points represents hue discriminability, with 0.1 being easily distinguishable. (*c*) The test phase consisted of five trials with different colours presented on the screen in a pseudorandom order (cue value: H, high; NH, near high; M, medium; NL, near low; L, low). In our example, the screen shows the medium colour with blue as the high-reward colour (H) and green as the low-reward colour (L), but this was counterbalanced across bees. Entering a chamber associated with a high reward or low reward during training was considered optimistic or pessimistic, respectively.

Visual stimuli were solid colours covering the entire display of an LED monitor (Dell U2412M, 24″, 1920 × 1200 px) and controlled by a custom-written MATLAB script (MathWorks Inc., Natick, MA, USA) using the PsychToolbox package [20]. We measured the irradiance of all colours used in the experiment using a spectrophotometer (Ocean Optics Inc., Florida, USA). The perceptual positions of the colours in the bee colour hexagon space (figure 1*b*) were calculated using the irradiance measurements and spectral sensitivity functions for *B. terrestris* photoreceptors [21,22].

We positioned two vertical panels (40.0 × 8.0 cm^2^) 8.5 cm in front of the right and left sides of the LED monitor, leaving the central area of the monitor open and visible. Each panel was equipped with an opening to place a reward chamber (7 ml glass vial, 10 mm inner diameter) 7 cm above the arena floor. After each visit to the arena, the reward chambers were changed to ensure pheromones and scent marks were not available during the next visit. In preparation for the next experimental day, all used chambers were washed in hot water and 70% ethanol and left to dry.

### (b) Training

Before the onset of training, individual bees were familiarized with both reward locations. A plastic cup was used to gently capture each bee. The opening of the cup was positioned to align with the entrance to the reward chamber, inside which the bee found a droplet of sucrose solution (0.2 ml, 30% w/w). We repeated the procedure equally on each side (left and right) without displaying any colour on the LED screen. Individual bees that learnt the reward location and performed repeated foraging bouts were tagged for later identification using number tags (Thorne, UK). Tagging involved trapping each bee in a marking cage, gently pressing it against the mesh with a sponge, and affixing the tag to the dorsal thorax with superglue (Loctite Super Glue Power Gel).

In each training trial, we presented bees (*n* = 48) one of two colours on the LED screen. The colours used were green (RGB = 0, 255, 75) and blue (RGB = 0, 75, 225). For a given bee, one of the colours (e.g. green) always indicated a low-value reward of 0.2 ml of 30% (w/w) sucrose solution in one of the two chambers (e.g. on the left), with the other chamber (e.g. on the right) containing an equal amount of distilled water. The other colour (e.g. blue) would be presented in different trials and would always indicate a high-value reward of 0.2 ml of 50% (w/w) sucrose solution in the chamber opposite (e.g. on the right) with the other chamber (e.g. on the left) containing an equal amount of distilled water. Thus, on any given trial, the bee saw only one colour and could encounter either the high or low reward (not both), with water on the unrewarding side. High and low rewards were always presented on opposite sides in their respective trials. The volume of reward chosen ensured that the bees were satiated with their visit and would return to the colony after they consumed it.

Across bees, the combinations of colour (green or blue), reward location (right or left) and reward type (high or low) were counterbalanced. Each bee encountered only one possible combination during training (e.g. green indicating a high reward on the left on half the trials, and blue indicating a low reward on the right on the other half). Trials presenting colours associated with high and low rewards were presented an equal number of times in a pseudorandom order, ensuring that no colour was repeated more than twice in a row. To ensure that the bee entered the reward chamber fully to sample its content, we placed the rewards at the end of the chamber. In all cases, the reward quantity allowed bees to fill their crop within a single visit [23]. We recorded a single choice on each trial, defined as a bee entering a chamber far enough to sample its content. Incidences of landing or partial entering (less than one-third of the body length) were not considered choices. These occurrences were rare and comprised only five out of all our choices. Bees that reached the learning criterion (80% accuracy in the last 20 trials) continued to the test phase. Eleven bees did not pass the initial conditioning test due to strong side biases. The last 10 training trials were video recorded using a camera on a mobile phone (Huawei Nexus 6P phone 1440 × 2560 px, 120 fps) placed above the arena. We calculated choice latency by averaging across latencies from the last two training trials of each respective colour.

### (c) Predatory attack simulation

Individual bees (*n* = 48 from 6 colonies) that reached the learning criterion in the training phase were randomly assigned to one of the three treatment groups, using the *sample* function in R. Two groups were subjected to manipulations simulating predatory attacks by shaking (*Shaking*, *n* = 16) or trapping (*Trapping*, *n* = 16). A third unmanipulated group served as a control (*Control*, *n* = 16). The manipulations were applied to a bee before entering the arena for each test. Bees in the control treatment were allowed to fly out into the flight arena without hindrance as in the training phase.

Each bee in the shaking treatment was allowed to enter a custom-made cylindrical cage (40 mm diameter, 7.5 cm length). After entering, the bee was gently nudged down with a soft foam plunger until the distance between the plunger and the bottom of the cage was reduced to ~3 cm. Once the plunger was secured, the cage with the bee was placed on a Vortex-T Genie 2 shaker (Scientific Industries, USA) and shaken at a frequency of 1200 r.p.m. for 60 s. After shaking, the bee was released into the tunnel connecting the nest box and experimental arena via an opening on the top of the tunnel. The bee was released into the flight arena for testing as soon as it was ready to initiate a foraging bout.

Each bee in the trapping treatment was trapped using a device similar to the robotic arm described in previous studies [7]. The mechanism consisted of a soft sponge (3.5 × 3.5 × 3.5 cm^3^) connected to a linear actuator system (rack and pinion). A micro-servo initiated the linear motion of the trapping device (Micro Servo 9 g, DF9GMS), powered and controlled by a microcontroller board (Arduino, Uno Rev 3). A custom-written script written in Arduino Software (IDE) triggered an initial plunging movement of the trapping device, followed by release after 3 s. This permitted consistent trapping across all individuals. The bee was then released into the flight arena for testing as soon as it was ready to initiate a foraging bout.

### (d) Judgement bias testing

The test phase consisted of five trials, each with a cue of a different colour presented on the screen. The test colours were the two conditioned colours (green and blue), and three ambiguous colours of intermediate value between the two conditioned colours (near blue (RGB = 0, 140, 150); medium (RGB = 0, 170, 120); near green (RGB = 0, 200, 100); figure 1*b*). We classified the ambiguous colours as near-high, medium and near-low cues depending on their distance to the high or low rewarding colour. The colour presentation order was pseudorandomized between all bees, so that the first test colour was always one of the ambiguous colour cues. During tests, all colour cues were not rewarded, i.e. both chambers contained 0.2 ml of distilled water. After the bee’s first choice, we gently captured it with a plastic cup and returned it to the tunnel connecting the nest and the arena. Between presentations of each of the test cues, bees were provided refresher trials consisting of two presentations of each conditioned colour with the appropriate reward at the correct location. All trials were recorded for video analysis using a mobile phone camera (Huawei Nexus 6P, 1440 × 2560 px, 120 fps). All experiments and video analyses were run by the experimenter with knowledge of the conditions.

### (e) Measuring feeding motivation

Stress can affect an animal’s feeding motivation as indicated by the amount of reward consumed [24]. To assess if our manipulations changed bee feeding motivation, we measured reward ingestion rates. A separate group of bees (*n* = 36) were pre-trained to forage from a feeder consisting of the reward chamber with a 1.5 ml Eppendorf placed inside. After learning this location and completing five consecutive foraging bouts, bees were randomly allocated to one of the three treatment groups for the ingestion test (*Control*: *n* = 12; *Shaking*: *n* = 12; *Trapping*: *n* = 12). The test consisted of a single foraging bout on a feeder with sucrose solution (~1 ml, 50% w/w). The feeder was weighed before and immediately after the test bout to determine the mass of ingested solution using a Kern Weighing Scale ADB100-4 (resolution: ±0.001 mg; Kern & Sohn, Balingen, Germany). Feeding bouts were recorded using a mobile phone camera (Huawei Nexus 6P, 1440 × 2560 px, 120 fps). The recordings were used to determine ingestion time, defined as the time from when the bee first touched the sucrose solution with its proboscis until the bee stopped drinking. For each bee, we calculated the absolute ingestion rate *i* (mg s^−1^):


i=(m1−m2)/t,

where *i* is the absolute ingestion rate of a bee, *m1* is the mass of the feeder before the foraging bout, *m2* is the mass of the feeder after the foraging bout and *t* is the ingestion time of the bee. After it completed the test, the bee was sacrificed by freezing and stored in 70% ethanol at −20°C. We measured the intertegular distance (*W*) and the length of the glossa of each bee with a digital calliper (RS PRO Digital Calliper, 0.01 mm ± 0.03 mm) under a dissecting microscope. We then adjusted the absolute ingestion rate *i* to account for individual size variability using the following formula:


$$I=iW^{\wedge }(1/3)G,$$

where *I* is the adjusted ingestion rate of a bee, *G* is the length of the glossa and *W* is the intertegular distance. This is an adaptation of the formula developed earlier [25] with intertegular distance instead of weight, as it has been shown to be precise at estimating bumblebee weights [26].

To control for evaporation, we placed an additional Eppendorf with 50% sugar solution on the opposite side of the test chamber and recorded its mass before and after each test. The loss of mass due to evaporation was subtracted from the mass of the solution after the foraging bout.

### (f) Video analysis

Video analysis was done using BORIS^©^ (Behavior Observation Research Interactive Software, v. 7.10.2107). In the judgment bias experiment, we coded two behaviours for each bee. The first, ‘Choice’, indicated bee entry into a reward chamber and was classified as a point event, an event which happens at a single point in time. The second behaviour, ‘Latency to choose’, was the time taken to choose and was classified as a state event, i.e. an ongoing event with a duration. For the foraging motivation experiment, we coded a single behaviour, ‘Drinking duration’, classified as a state event indicating ingestion time.

### (g) Statistical analysis

Our hypothesis and statistical analyses of the main active choice experiment were preregistered at aspredicted.com (62198). The data were plotted and analysed using RStudio v. 3.2.2 (R Foundation for Statistical Computing, Vienna, Austria). To determine the final sample size needed, we used a Bayes factor approach implemented with the brms package (see electronic supplementary material for details) [27–29]. Subsequent statistical models were fitted by maximum-likelihood estimation and, when necessary, optimized with the iterative algorithms [30]. Models were compared using the model.sel function in the MuMIn package [31] and the model with the lowest Akaike information criterion (AIC) score was selected as the best model. We used the package DHARMa [32] for residual testing of all models.

For the judgment bias analysis, we used the probability of choosing the chamber associated with a high reward as the dependent variable, coding choices of reward chambers previously associated with high- and low-value cues as 1 and 0, respectively. For ease of discussion, we henceforth call the choices of high-reward chambers ‘optimistic’ and choices of low-reward chambers ‘pessimistic’. We fitted a generalized linear mixed-effect model using the *glmer* function of the *lme4* package with binomial errors and a logit link function. The explanatory variables included in the model were ‘*Treatment*’ (categorical: *Control*, *Shaken*, *Trapped*) and ‘*Cue*’ (continuous: 1–5, where 1 = high and 5 = low value cue) which refers to the colour displayed on the screen. The identity of the bee (‘*ID*’) was included as a random intercept variable.

To analyse choice latency, we fitted a linear mixed-effect model (LMEM) using the *lmer* function of the *lme4* package. Latency data were log-transformed and latencies greater than 1.5 times the interquartile range were excluded (a total of 18 out of 240 data points). The explanatory variables included in the model were ‘*Treatment*’ (categorical: *Control*, *Shaken*, *Trapped*) and ‘*Cue*’ (continuous: 1–5, where 1 = high and 5 = low value cue). In addition, since we expected optimistic responses to be faster, we included ‘*Response Type*’ (coded as 1 and 0 for optimistic and pessimistic responses, respectively) as an explanatory variable. Bee identity (‘*ID*’) was included as a random intercept variable.

Data for other analyses were first tested for normality before using appropriate tests. We ran a one-way ANOVA on the body-size-adjusted ingestion rate to test for treatment differences. We also used Kruskal–Wallis tests to compare the average number of trials to the criterion in the training phase across treatments, and to investigate the impact of the side and colour associated with a high-value cue.

### (h) Signal detection theory model

We examined whether the behaviour of the bees could be modelled with standard signal detection theory [33], and what we could infer about the underlying mechanisms. We assumed that bees learn to make their foraging decision during training based on the value of an internal signal *x* which indicates whether they are in a high- or low-reward situation. We specified *x* as a ‘low-reward signal’ with a high value when the cue indicates a low reward. We assumed that bees have some internal decision boundary *B*, such that when *x* > *B*, they behave appropriately for the low-reward situation, and conversely when *x* < *B* for the high-reward situation. Although on average the value of *x* reflects the cue, it is affected by noise, explaining why bees do not always make the same decision in the same experimental situation.

Since we have fitted our data with a logistic link function, we modelled the distribution of the noisy signal as the first derivative of a logistic function. This allowed our signal detection model to predict logistic response curves, as we see below. The standard logistic is


(2.1) $$F\left(x\right)=\frac{1}{1+\exp\left(-x\right)},$$

and its first derivative is


(2.2) $$f\left(x\right)=\frac{dF}{dx}=\frac{\exp\left(x\right)}{{\left[1+\exp\left(x\right)\right]}^{2}},$$

which is therefore the distribution we assume for our noise.

The probability density function governing the distribution of the signal *x* is therefore $\frac{1}{\sigma }f\left(\frac{x-C}{\sigma }\right),$ where *C* represents the value of the cue and σ is the standard deviation of the noise. The probability of an optimistic response on any given trial is the probability that the value of *x* on this trial is less than the decision boundary *B*, given the value of the cue on this trial. This is


(2.3) $$P_{opt}=\int_{-\infty }^{B}dx\frac{1}{\sigma }f\left(\frac{x-C}{\sigma }\right)=F\left(\frac{B-C}{\sigma }\right).$$

As noted above, with the assumption that the noise distribution is the logistic-derivative, *f*(*x*), the probability of an optimistic response is a logistic function of cue *C*.

As well as the cue, the bee’s behaviour is influenced by the noise *σ* and the decision boundary *B*. The noise may vary depending on factors like fatigue or attention, while the decision boundary may reflect a cognitive strategy. A common assumption is that the decision boundary is chosen to maximize expected reward. We therefore calculated the expected reward during training.

On trials where the cue *C* was set to *C*_Hi_, optimistic responses are made with probability $F\left(\frac{B-C_{Hi}}{\sigma }\right)$ and rewarded with 50% sucrose, with perceived value denoted as *R*_Hi_. Conversely, pessimistic responses are made with probability $\left[1-F\left(\frac{B-C_{Hi}}{\sigma }\right)\right]$ and obtain only water, of value *R*_w_. The average reward experienced on high-value-cue trials is thus


$$<R>{|}_{C=C_{Hi}}=\;R_{Hi}F\left(\frac{B-C_{Hi}}{\sigma }\right)+R_{w}\left[1-F\left(\frac{B-C_{Hi}}{\sigma }\right)\right].$$

On trials where the cue *C* was *C*_Lo_, optimistic responses are made with probability $F\left(\frac{B-C_{Lo}}{\sigma }\right)$ and result in water, *R*_w_, whereas pessimistic responses are made with probability $\left[1-F\left(\frac{B-C_{Lo}}{\sigma }\right)\right]$ and rewarded with 30% sucrose*, R*_Lo_. The average reward on low-value-cue trials is thus


$$<R>{|}_{C=C_{Lo}}=R_{w}F\left(\frac{B-C_{Lo}}{\sigma }\right)+R_{Lo}\left[1-F\left(\frac{B-C_{Lo}}{\sigma }\right)\right].$$

Overall, then, the expected reward during training is


(2.4) $$<R>\;=P_{Hi}R_{Hi}F\left(\frac{B-C_{Hi}}{\sigma }\right)+P_{Hi}R_{w}\left[1-F\left(\frac{B-C_{Hi}}{\sigma }\right)\right]+P_{Lo}R_{Lo}\left[1-F\left(\frac{B-C_{Lo}}{\sigma }\right)\right]+P_{Lo}R_{w}F\left(\frac{B-C_{Lo}}{\sigma }\right),$$

where *P*_Hi_ and *P*_Lo_ represent the probabilities that a given trial offers high or low reward.

The optimal boundary *B*_opt_ that maximizes the expected reward then satisfies the equation


(2.5) $$P_{Hi}\left(R_{Hi}-R_{w}\right)f\left(\frac{B_{opt}-C_{Hi}}{\sigma }\right)=P_{Lo}\left(R_{Lo}-R_{w}\right)f\left(\frac{B_{opt}-C_{Lo}}{\sigma }\right)$$

(found by taking the derivative of the expected reward, equation (2.4), with respect to *B* and finding where this is equal to 0).

Equation (2.5) has a simple graphical interpretation (see fitted model in figure 3). First, the probability distributions for high and low reward are rescaled by their estimated probability and by the additional utility of getting the trial right, compared with the water available with the wrong decision. Then, the optimal boundary is where these rescaled distributions cross over (solid vertical lines in figure 3). If the cue probabilities and reward utilities were equal, i.e. $P_{Hi}\left(R_{Hi}-R_{w}\right)=P_{Lo}\left(R_{Lo}-R_{w}\right)$ , then the optimal decision boundary would be exactly in the middle between the two cue values: $B_{opt}=0.5\left(C_{Hi}+C_{Lo}\right)$ .

### (i) Drift diffusion model

Drift diffusion models shed light on the cognitive processes underlying decision-making in choice tasks [34]. They generate estimates of the time taken to accumulate sensory evidence for a particular response and the evidentiary threshold for the response decision. We used this framework to investigate which of these two criteria (or both) were changed due to our stress manipulations.

We fitted a drift diffusion model to the choice latency data in our three treatments using the R package rtdists [35]. The model assumes that the bee accumulates sensory evidence towards a decision and makes the optimistic or pessimistic choice once the evidence has passed a threshold. Pessimistic and optimistic choice thresholds were defined to be at 0 and 1, respectively. The decision variable was assumed to begin from a start point *z* between the two boundaries. It was subject to random noise represented by the diffusion constant *s* but had a drift rate *v* towards one or the other boundary, based on the sensory evidence. In our experiment, *v* should be positive for Cue = 1 and negative for Cue = 5. In our model, we assumed that *v* was a linear function of Cue.

## 3. Results

### (a) Training

During training, 48 bumblebees achieved the learning criterion and continued to the judgment bias test. There were no significant differences in the number of trials required to reach the criterion among bees that experienced the high reward on the right or left location (Kruskal–Wallis test: χ^2^ = 2.94, d.f. = 1, *p* = 0.09). Similarly, there was no significant difference in the total number of trials to criterion for bees experiencing blue or green as the high reward colour (Kruskal–Wallis test: χ^2^ = 0.94, d.f. = 1, *p* = 0.33). The number of trials to criterion also did not differ among bees used in each of the three treatment groups (Kruskal–Wallis test: χ^2^ = 0.88, d.f. = 2, *p* = 0.64).

Bees took significantly longer to choose a low reward compared with a high reward in the last choices of training (electronic supplementary material, table S2; LMEM, estimate ± s.e. = 0.59 ± 0.09, *t* = 6.79, *p* < 0.001). The difference in latencies demonstrates that the bees could differentiate between both the colour cues and the two rewards.

### (b) Physically stressed bees are less optimistic

**Figure 2.**
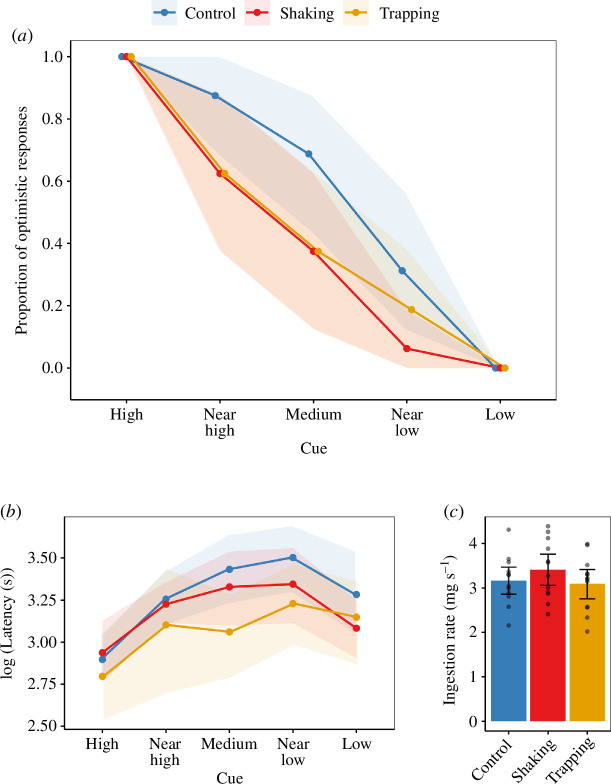
Bee responses to test cues. (*a*) Proportion of bees (*n* = 16 per treatment) making an optimistic choice (choosing a reward chamber associated with high reward) in response to each of five cues. (*b*) Response latency to each of five cue values (*n* = 16 bees per treatment). (*c*) Average ingestion rate of high reward (50% sugar solution) for bees in each treatment group (*n* = 12 bees per treatment). The treatment groups were control (blue), shaking (red) and trapping (orange). The test cues were high, near high, medium, near low and low value cues depending on their distance to the colours of high- and low-reward cues. Points and bars represent means. Shaded areas and error bars represent 95% bootstrapped confidence intervals. Dots represent values from individual bees.

The best model for our data included main effects of cue colour and treatment but no interaction effect (see electronic supplementary material, table S1, for model selection). Shaking significantly reduced the probability of optimistic responses, i.e. choosing the location associated with a high reward (figure 2*a*; electronic supplementary material, table S2; generalized linear mixed model (GLMM), estimate ± s.e. = −1.49 ± 0.57, *z* = −2.61, *p* < 0.01). Trapping also significantly reduced the likelihood of an optimistic response (figure 2*a*; electronic supplementary material, table S2; GLMM, estimate ± s.e. = −1.26 ± 0.56, *z* = −2.23, *p* = 0.026). Bees were also significantly less likely to respond optimistically to cues with colours further away from that of the high-reward cue (figure 2*a*; electronic supplementary material, table S2; GLMM, estimate ± s.e. = −1.79 ± 0.21, *z* = −8.39, *p* < 0.001). All bees always made a choice, i.e. bees not responding optimistically responded pessimistically.

### (c) Choice latencies and feeding motivation

The best-fitting model for choice latency during tests included treatment, cue value and response type (optimistic or pessimistic) as fixed predictors and an interaction between cue value and response type (electronic supplementary material, table S1). Bees in the trapping treatment were significantly faster to make a choice than control bees (figure 2*b*; electronic supplementary material, table S2; LMEM, estimate ± s.e. = −0.23 ± 0.1, *t* value = −2.25, *p* = 0.029). Shaken bees were not significantly faster to make their choices than control bees (figure 2*b*; electronic supplementary material, table S2; LMEM, estimate ± s.e. = −0.11 ± 0.10, *t* value = −1.121, *p* = 0.27). All bees were significantly slower to make a choice when the cue colour was further away from that of the high-reward cue (LMEM, estimate ± s.e. = −0.09 ± 0.03, *t* value = −2.6, *p* < 0.01). Bees were faster when making optimistic choices compared to pessimistic ones (LMEM, estimate ± s.e. = −0.93 ± 0.16, *t* = −5.74, *p* < 0.001). Additionally, a significant interaction between cue value and response type (LMEM, estimate ± s.e. = 0.262 ± 0.051, *p* < 0.001) indicated that the decrease in latency with increasing cue value was more pronounced for optimistic responses.

The mean ingestion rates in our feeding motivation experiment did not differ significantly between treatment groups (figure 2*c*; ANOVA: *F*(2, 33) = 0.881, *p* = 0.424).

### (d) Signal detection theory model

According to a standard signal-detection theoretic approach, the probability that a bee makes an optimistic choice for cue level *C* is (equation (2.3))


$$P_{opt}=F\left(\frac{B-C}{\sigma }\right),$$

where *σ* is the noise on the internal signal, *B* is the decision boundary and *F* is the logistic function. This is exactly the model fitted by our GLMM (see above), with the fitted gradient for *Cue* corresponding to -1/σ and the intercept corresponding to B/σ . Thus, the fact that we found no interaction between *Cue* and *Treatment* suggests that the effective noise level is not changed by our manipulations. The estimate of −1.79 for the gradient (electronic supplementary material, table S2) allows us to infer an effective noise level of *σ* = 0.56, in our units where *Cue* runs from 1 (high reward) to 5 (low reward).

**Figure 3.**
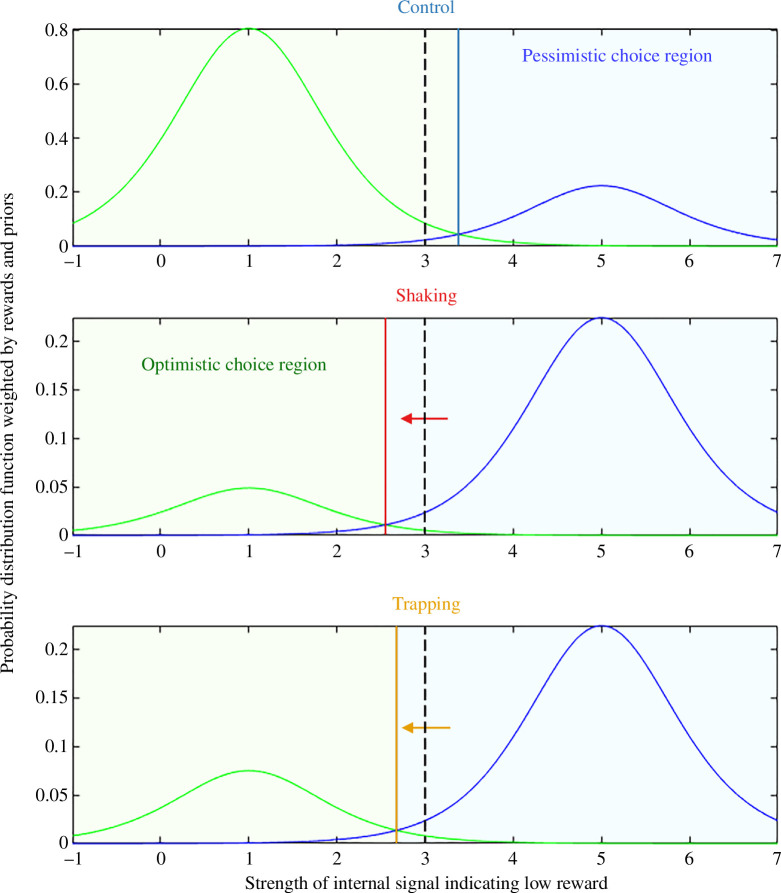
Bee decision-making boundaries and priors fitted by a signal-detection model. Curves depict the probability density functions for responses based on the internal signal *x* indicating a low reward. In each case, the original distribution has been weighted by the product of the value of that reward and its probability of occurring (see equation (2.5)). The two curves in each panel depict the probabilities that the cue indicates high reward (green, centred on 1) or low reward (blue, centred on 5). Solid lines depict the decision boundary *B* inferred from the model fit to our data. Dotted lines indicate the medium point for comparison. Regions to the right of the solid boundary line are regions where the bee makes pessimistic choices (shaded blue). Regions to the left are regions where the bee makes optimistic choices (shaded green). Arrows depict the shift in boundaries compared to the control condition. The three panels depict the conditions for the Control (top), Shaking (middle) and Trapping (bottom) treatments. Note the change in axes in the lower two panels.

However, the significant main effect of *Treatment* indicates that the decision boundary was different in the two cases. The estimate of 6.05 (electronic supplementary material, table S2) for the intercept in the control condition implies that the decision boundary in this condition is 3.38. Bees in the control treatment are thus equally likely to make optimistic or pessimistic responses when the cue is a little closer to ‘near low’ than medium (3). The fact that the intercept drops by −1.49 for the shaking treatment and −1.26 for trapping (electronic supplementary material, table S2) implies that the boundary shifts leftward to 2.55 and 2.68, respectively, in these conditions. The point at which these bees are equally likely to make optimistic and pessimistic choices is closer to ‘near high’ than to medium (figure 3).

In our fitted model, weighted probability distributions for both low and high rewards have an equal spread, reflecting the noise level inferred from the GLMM. In the control treatment, the shift of the decision boundary reflects the greater weight given to the high reward. Quantitatively, the extent of the shift, together with the fitted noise level, implies that the high reward is given 3.6 times the weight of the low reward. This result cannot be explained merely by the bees not perceiving the medium colour as midway between blue and green since both the high and low reward trials combine data from trials where the cue was blue and trials where it was green. Instead, this result might, for example, suggest that the bees understand that both rewards are equally likely (*P*_Hi_ = 50%) and find the 50% sucrose solution 3.6 times as rewarding, relative to water, as the 30% solution.

The fact that the decision boundary is to the left of neutral in the shaking and trapping treatments suggests that here, greater weight is given to the low reward (figure 3). Assuming we can discount the possibility that the reward value has inverted (i.e. that stressed bees find 30% sucrose more rewarding than 50%), this must represent a shift in their estimates of reward probabilities, such that stressed bees now consider high-reward trials less likely. To match the extent of the leftward shift, given the noise level inferred from our GLMM fit, the low reward must be weighted 4.6 times as much as the high reward. If the reward ratio were 3.6, this would imply that the bees behave as if the perceived probability of the high reward was 6%. However, if stressed bees find 50 and 30% sucrose equally valuable, i.e. the stress has removed the difference in reward utility, then the observed shift in decision boundary could be produced with a less dramatic shift in estimated probability, with perceived probability of the high reward being 18%.

### (e) Drift diffusion model

**Figure 4.**
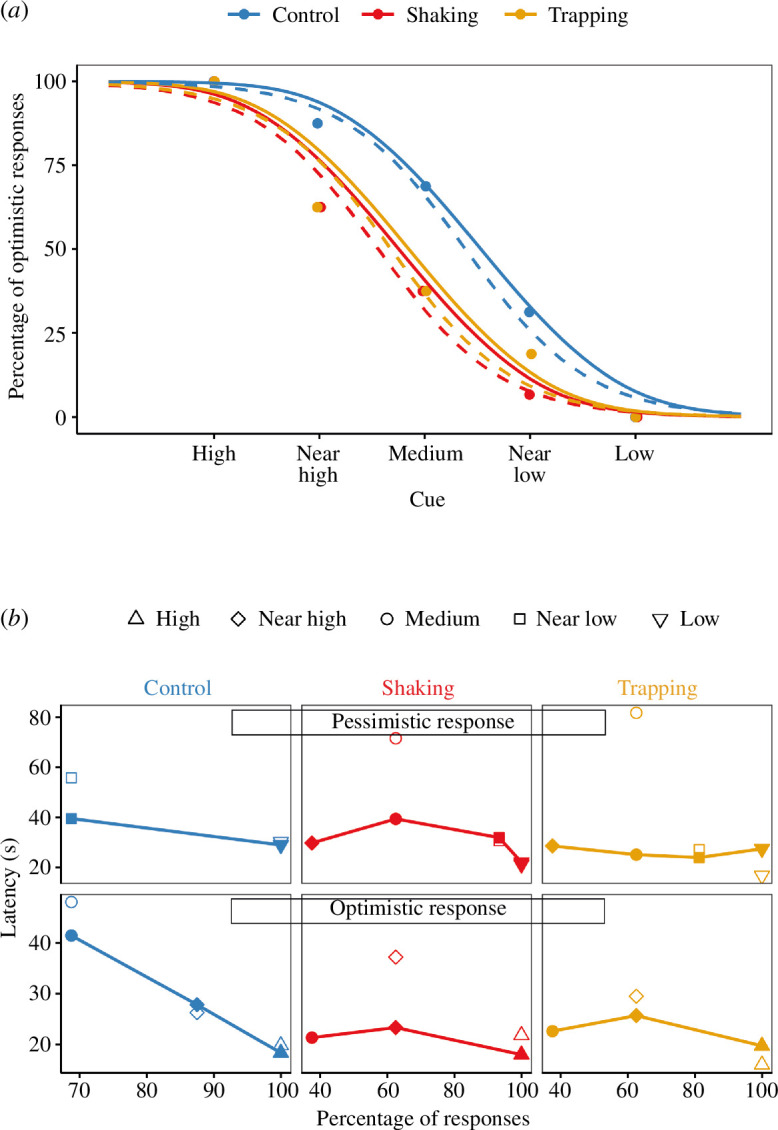
Drift diffusion model. (*a*) Proportion of optimistic choices made by the bees in each treatment in response to the different cues. Points show the data, dashed curves show the predictions of a fitted logistic regression model with main effects of *Treatment* and *Cue* but no interaction. Solid curves show predictions of a fitted drift diffusion model. Colours depict the different treatments: Control (blue), Shaking (red) and Trapping (orange). (*b*) Drift diffusion model fit to latencies. Filled symbols linked with lines show median latencies as a function of the percentage of responses made for pessimistic (top) and optimistic (bottom) responses in the three treatments (columns). Open symbols show predictions of the fitted drift diffusion model. Symbols show *Cue* value. There is a high percentage of optimistic responses for high (triangles) and near high (diamonds) cues and a high proportion of pessimistic responses for low (inverted triangles) and near low (squares) cues.

Our best model was obtained by allowing the time before making a decision and the value of the drift rate for *Cue* = 3 (v3) to vary between treatments, while fitting all data with the same values for the diffusion constant *s*, start point *zr*, the dependence of drift rate on cue, *vGradient* and noise on the drift rate, *sv*. The drift diffusion model predicts not only the bees’ choices (figure 4*a*) but also the latencies for both optimistic and pessimistic choices (figure 4*b*). There are not enough trials to accurately estimate the latency distributions (just 16 trials for each *Cue/Treatment* combination, thus <16 for each choice). The model for latencies is, therefore, not a good fit (figure 4*b*). With that caveat, the fitted model implies a few key points.

Firstly, by default, bees tend to be biased towards the more rewarding choice. The start point for the decision variable is not midway between the two boundaries, 0.5, but closer to the boundary for the optimistic choice, 0.56. Secondly, stress did not affect sensory noise. We found that the best model was again obtained by assuming that sensory noise was the same for all groups. Thirdly, stressed bees spend less time on non-decision activity: the model fitted more time on non-decision activity (e.g. flying across the arena) for the control bees than for the shaken or trapped bees. This could perhaps suggest that stressed bees might not want to spend time exploring what could potentially be a dangerous environment. Finally, this model also confirms that the stressed bees are more pessimistic. This is shown by the fitted drift rate for the medium cue, *Cue* = 3. In the absence of bias, the drift rate should have been zero in this case since the cue was designed to be exactly midway between the high- and low-reward cues. Control bees nevertheless showed a small positive drift rate for this cue, indicating that they took it as weak evidence for high reward. However, shaken and trapped bees both showed a small negative drift rate, indicating perceived weak evidence for low reward. This is what accounts for the leftward shift in the response curves for stressed bees. Note that even though, according to the model, all bees start slightly biased towards a high-reward response (*z* = 0.55), in stressed bees, the negative drift rate for the medium cue is enough to bias responses towards the pessimistic response.

## 4. Discussion

Our results show that in response to ambiguous cues, stressed bees were less likely than control bees to choose locations that were previously high rewarding. Our models suggest that this is due to a reduced estimate of the probability of high rewards.

Most studies of judgment bias use a go/no-go paradigm. The results of these studies can be challenging to interpret due to confounds from other factors that do not involve stimulus judgements such as motivation [19]. Our active choice design avoids these complications, so motivation alone cannot explain the observed shift in responses. This is further supported by our ingestion rate experiment, which shows no differences in feeding motivation. Furthermore, in one previous test of insect judgment biases, shaken honeybees showed a decreased proportion of ‘go’ responses not only to ambiguous odour mixtures but also to the conditioned negative odour [8]. This decrease could indicate an improved ability to differentiate odours rather than a negative bias in judgement [36]. In our experiment, however, the bees were perfectly accurate when responding to both conditioned cues in the tests. Our manipulations thus did not impair the colour discrimination abilities and memory of the bees.

Response latencies in judgement bias tests can be particularly difficult to interpret. For instance, exposure to a positive event has been reported to cause both longer [37] and shorter [38] response times to ambiguous stimuli. Increased latencies may also be associated with a general increase in reactivity and arousal, due to, say, the increased energetic demands of stressful events [39]. It may also indicate a shift in the perceived value of the reward and differences in motivation [40]. Shorter latencies to ambiguous cues, on the other hand, could result from factors like neophobia rather than negative interpretations of those cues [41].

Only one study has used latencies to measure judgment biases in bees [7]. This study demonstrated an optimistic bias in bumblebees, showing that unexpected sugar solution rewards reduced the latency with which bees approached ambiguous stimuli. However, the treatment also caused an increase in thoracic temperature which has been linked to increased foraging motivation [42]. Despite the study’s controls, motivation and arousal alone could potentially explain these results [19]. In our study, trapped bees had shorter latencies than control bees. Based on the approach in the previous study, this could suggest an optimistic bias. However, this interpretation would be misleading, as changes in feeding motivation or general arousal can also cause faster latencies. While arousal is widely used to characterize emotional states, both positive and negative states can involve increased arousal levels [43]. Our design allows us to more reliably use active choices to indicate affective valence. In the absence of active choices, it is difficult to determine whether increased approach latencies indicate changes in emotional valence or merely changes in motivation. It is also important to note that our different treatments kept the bee out of the colony for differing amounts of time. This could additionally contribute to stress levels and have an influence on response latencies.

One previous study has used an active choice design to study judgement biases in insects [10]. In that study, flies were trained to associate two odours, with either a reward or a punishment. Our study instead uses rewards of different quality, allowing us to investigate how states modulate expectations and perceptions of reward. Using paradigms involving reward and punishment can make it easier to detect affect-dependent judgement bias compared to paradigms with two rewards [14]. Therefore, finding a bias using two rewards, as we do, provides robust evidence for affect-dependent processing in insects.

Measuring active choices also allowed us to use a signal detection approach. This has been suggested as a valuable tool for investigating affective disorders but has rarely been applied in human clinical studies [44]. A recent study suggested that judgement biases in bees may be caused by a shift in stimulus–response gradients [11]. However, this study did not investigate the underlying cognitive mechanisms of this shift. In our model, the estimation of future outcomes combines estimates of the probability of an outcome and the magnitude of the pay-off from an outcome. Our models demonstrate that control bees respond more optimistically to ambiguous cues, indicating an expectation of high rewards. Such a bias would in fact be what is predicted by a rational, fully informed strategy which optimizes expected reward. Even if the bees are estimating the probabilities correctly as 50–50, the difference in reward utility will still shift the decision boundary towards the cue indicating low reward (figure 3).

The decision boundary and drift rate for the stressed bees are harder to interpret. Here, the decision boundary is to the left of neutral and the drift rate is negative. Previous studies have shown that acute stress can increase an animal’s sensitivity to the reward [45]. However, the observed left shift of the decision boundary in stressed bees cannot plausibly reflect such a change in sensitivity since a leftward shift could only be produced if the values of high and low rewards were swapped, i.e. if 50% sucrose became less rewarding than 30%. However, it could reflect a pessimistic bias in expectations, i.e. that the stressed bees behave as if high rewards are less likely (*P*_Hi_ < *P*_Lo_). This can account for a leftward shift, but the large quantitative extent of the shift is still surprising. Since the noise remains relatively small, as indicated by the perfect performance for high and low cues, we have to postulate enormous changes in the priors to produce the observed shift. To obtain the decision boundary of 2.55 inferred for shaken bees, we would have to postulate that shaken bees estimate *P*_Lo_ = 94%, i.e. they expect a high reward to be available on only one trial in 20. This assumes that the reward utility remains the same. If the relative utility of the high reward increased, e.g. because of an increased need for sucrose after stress [39], the estimated probabilities would have to shift even further from 50%. However, one possibility is that, contrary to the assumptions of our model, the noise was not uniform for all cues, and there was more sensory noise on intermediate values of the cue. If so, the change in probabilities would not need to be as dramatic, although the basic result of changed probabilities would remain true.

Could the pessimistic judgements of the bees be adaptive? Emotions have evolved to guide behaviour by informing animals about their success in obtaining resources and avoiding dangers in their environment [43]. Pessimism, for example, could be an adaptive strategy in a dangerous and unpredictable environment [46]. A pessimist is more likely to avoid risky decisions that could jeopardize gains in pursuit of more rewarding opportunities, which, in unfavourable environments, could be unlikely. In our study, stressed bees experience a simulated predatory attack. This could exhaust their energetic stores and signal a dangerous environment. In response to the attack, the bees lowered their reward expectations. This could reflect more cautious behaviour, a potentially adaptive strategy in a dangerous environment. Our results thus suggest the possibility of shared adaptive responses across diverse taxa.

While the present study further validates the capacity of bees for emotion-like states, the mechanisms behind these behaviours remain largely unknown. Future research should focus on understanding how these states are generated and sustained. Clarifying these mechanisms will help determine whether the observed states share a common ancestry with similar states in vertebrates or are distinct and result from convergent evolution.

## Data Availability

All relevant data and code used for analysis to support this paper are available as electronic supplementary material [47].
